# Gait Event Detection for Stroke Patients during Robot-Assisted Gait Training

**DOI:** 10.3390/s20123399

**Published:** 2020-06-16

**Authors:** Andreas Schicketmueller, Juliane Lamprecht, Marc Hofmann, Michael Sailer, Georg Rose

**Affiliations:** 1 HASOMED GmbH, Paul-Ecke-Str. 1, 39114 Magdeburg, Germany; marc.hofmann@hasomed.de; 2 Institute for Medical Engineering and Research Campus STIMULATE, University of Magdeburg, Universitaetsplatz 2, 39106 Magdeburg, Germany; Georg.Rose@ovgu.de; 3 MEDIAN Neurological Rehabilitation Center Magdeburg, Gustav-Ricker-Str. 4, 39120 Magdeburg, Germany; Juliane.Lamprecht@median-kliniken.de (J.L.); michael.sailer@median-kliniken.de (M.S.); 4 Institute for Neurorehabilitation, Affiliated Institute of the Otto-von-Guericke University, Gustav-Ricker-Str. 4, 39120 Magdeburg, Germany; 5 MEDIAN Clinic Flechtingen, Parkstraße, 39345 Flechtingen, Germany

**Keywords:** stroke, inertial measurement unit (IMU), functional electrical stimulation (FES), gait event detection, hybrid robotic rehabilitation system

## Abstract

Functional electrical stimulation and robot-assisted gait training are techniques which are used in a clinical routine to enhance the rehabilitation process of stroke patients. By combining these technologies, therapy effects could be further improved and the rehabilitation process can be supported. In order to combine these technologies, a novel algorithm was developed, which aims to extract gait events based on movement data recorded with inertial measurement units. In perspective, the extracted gait events can be used to trigger functional electrical stimulation during robot-assisted gait training. This approach offers the possibility of equipping a broad range of potential robot-assisted gait trainers with functional electrical stimulation. In particular, the aim of this study was to test the robustness of the previously developed algorithm in a clinical setting with patients who suffered a stroke. A total amount of N = 10 stroke patients participated in the study, with written consent. The patients were assigned to two different robot-assisted gait trainers (Lyra and Lokomat) according to their performance level, resulting in five recording sessions for each gait-trainer. A previously developed algorithm was applied and further optimized in order to extract the gait events. A mean detection rate across all patients of 95.8% ± 7.5% for the Lyra and 98.7% ± 2.6% for the Lokomat was achieved. The mean type 1 error across all patients was 1.0% ± 2.0% for the Lyra and 0.9% ± 2.3% for the Lokomat. As a result, the developed algorithm was robust against patient specific movements, and provided promising results for the further development of a technique that can detect gait events during robot-assisted gait training, with the future aim to trigger functional electrical stimulation.

## 1. Introduction

According to the WHO Global Health Estimates, cerebrovascular accidents such as stroke are the second leading cause of death and the third leading cause of disability worldwide [1]. Amongst other pathologies, walking dysfunction is a major problem for many subjects who have suffered a stroke [2,3]. Furthermore, walking dysfunctions can lead to falls and restrict the patient in performing the activities of daily living. Thus, the improvement of independent walking is a primary goal of stroke rehabilitation [4]. Besides conventional physiotherapy, robot-assisted gait training is used to counteract gait disorders. In a clinical routine, robots such as the Lokomat (Hocoma, Volketswil, Switzerland), Gait Trainer GT II (Reha Stim, Berlin, Germany), Lyra (Thera Trainer, Hochdorf, Germany) and G-EO (Reha Technology AG, Olten, Switzerland) are used. The benefits of these robots are twofold; the bodyweight support system of the robot carries the weight of the patient, and the gait pattern is induced by a robot specific strategy which leads to physical relief for the therapists. Additionally, patients who receive electromechanical-assisted gait training in combination with physiotherapy after stroke are more likely to achieve independent walking than patients receiving gait training without these devices [5]. Besides the positive effects of robot-assisted gait training, there are also drawbacks of this technology. The bodyweight support system partially inhibits muscle activity [6], and limited degrees of freedom of leg and pelvis movement can lead to changes in the naturally occurring muscle activation patterns [7]. These drawbacks are tackled by developments of the aforementioned devices. Robot specific strategies, such as the biofeedback mode of the Lyra, the active-assistive/active mode of the G-EO or the alteration of the guidance force and FreeD mode of Lokomat, aim to improve the rehabilitation by allowing the subject to actively influence the robot-assisted gait training using their remaining muscle activity. Another approach to enhance rehabilitation is the combination of robot-assisted gait training and functional electrical stimulation (FES). The combination of these techniques is termed a hybrid robotic rehabilitation system, which has been shown to be more effective than robot-assisted gait training alone [8]. Approaches to combine systems like Lokomat or the GaitTrainer GT II with FES were promising [9,10]. The G-EO, and the RT600 (Restorative Therapies, Baltimore, USA) system are commercially available hybrid robotic rehabilitation systems which can provide additional FES treatment during gait training. However, most of these systems depend on information (gait events) provided by the robot [10,11], focus on stimulating with intramuscular electrodes [12], or focus on foot drop with a limited number of stimulated muscles [9]. In order to extend the application and the use of robot-assisted rehabilitation, especially with FES, a novel algorithm was developed. In perspective, the aim is to combine inertial measurement units (IMUs) and robot-assisted gait training to trigger FES [13]. This approach was feasible in a clinical setting, and was tested in a first step with a healthy subject. Thus, the objective of this study was to examine the robustness of the algorithm when used in a clinical setting with stroke patients, and to implement potential improvements.

## 2. Materials and Methods

### 2.1. Subjects

**Figure 1:**
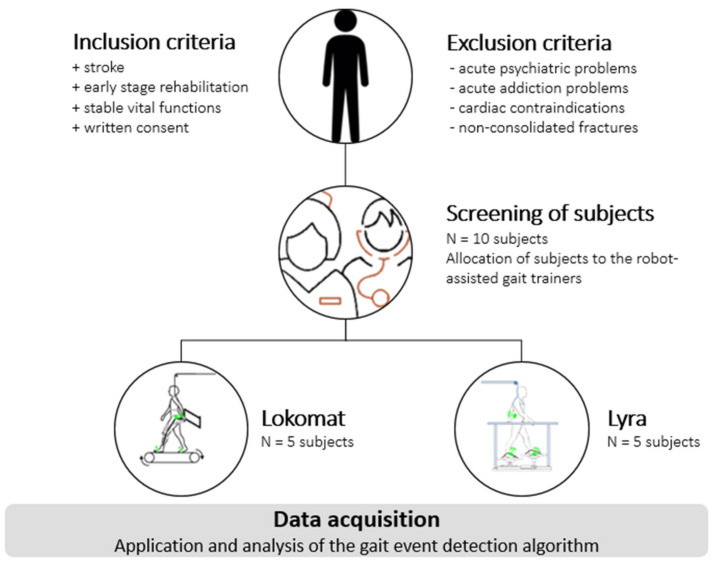
Study procedure.

A total amount of N = 10 stroke patients, with written consent, participated in the study. The subject recruitment was carried out in the MEDIAN Neurological Rehabilitation Center, Magdeburg. To determine the subject’s suitability to robot-assisted gait training, all patients were screened by specially trained therapists. The patients were assigned to the robots according to their performance level. The according study procedure can be seen in Figure 1. Since therapists coordinate the training and aim for the best rehabilitation results, the training velocity of the gait trainer (Section 2.3) was not predetermined, but was adjusted according to the need of the subject during the therapy. The training was limited to level-ground walking, as the robot-assisted gait trainers used (Section 2.3) do not provide other typical gait patterns, such as backwards walking, stairs, or slope climbing (ascending and descending). Additionally, the subjects were new to robot-assisted gait training; thus, the recommended treatment plan indicated level-ground walking. The therapy time for each individual was 90 min, including the screening of the patient, setup time, explanation of the study procedure and robot-assisted gait training. The measurements were realized during the normal daily routine of the clinic. Thus, a normal rehabilitation procedure of the participating subject was guaranteed.

### 2.2. Inertial Measurement Units

**Figure 2:**
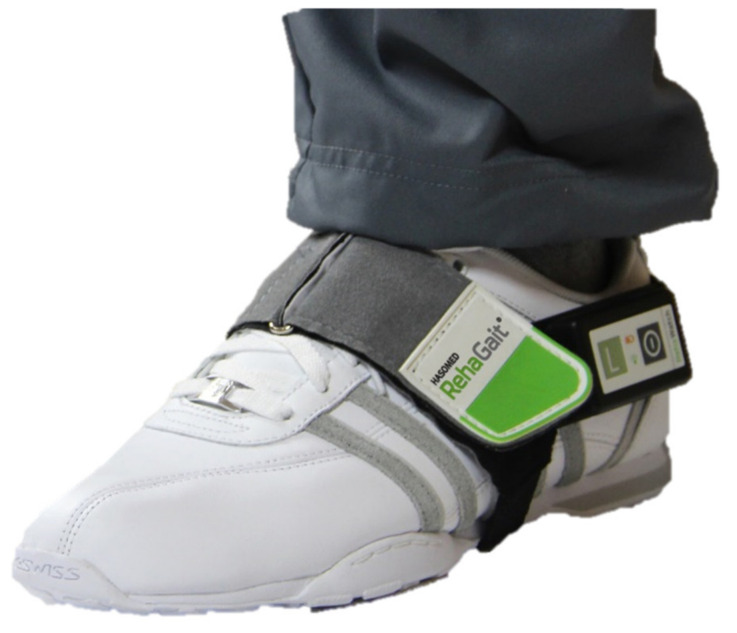
Inertial measurement unit with fixation strap.

Two inertial measurement units (MotionSensor, HASOMED GmbH, Magdeburg, Germany) were used for data acquisition. Each IMU consisted of a three-axis accelerometer (±16 g) and a three-axis gyroscope (±2000 °/s) [14]. The IMUs were attached to the subject’s foot using custom-built fixation straps (Figure 2), and the sampling frequency was set to 500 Hz.

### 2.3. Robot-Assisted Gait Trainers

**Figure 3:**
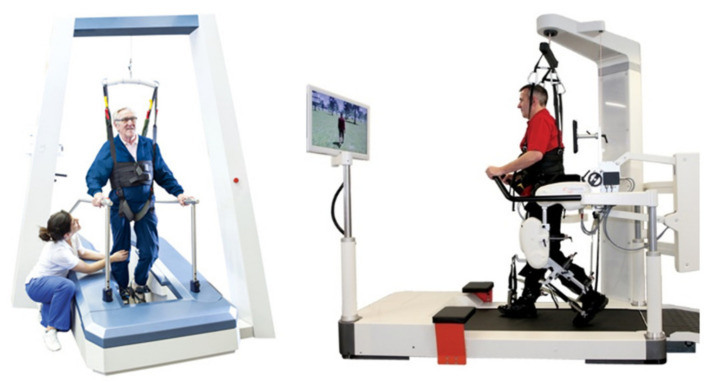
Robot-assisted gait trainer. Lyra (Thera Trainer, Hochdorf, Germany) (left); Lokomat (Hocoma, Volketswil, Switzerland) (right).

Robot-assisted gait trainers can be categorized according to their movement principle into two groups; exoskeletons and controllers of endpoint trajectories [15]. Exoskeleton robots, such as the Lokomat (Figure 3—right image), move knee and ankle joints during the gait training, whereas controllers of endpoint trajectories, such as the Lyra (Figure 3—left image), move the legs using adjustable foot plates. Both principles have been shown to provide good rehabilitation results [5,16].

### 2.4. Experimental Setup

**Figure 4:**
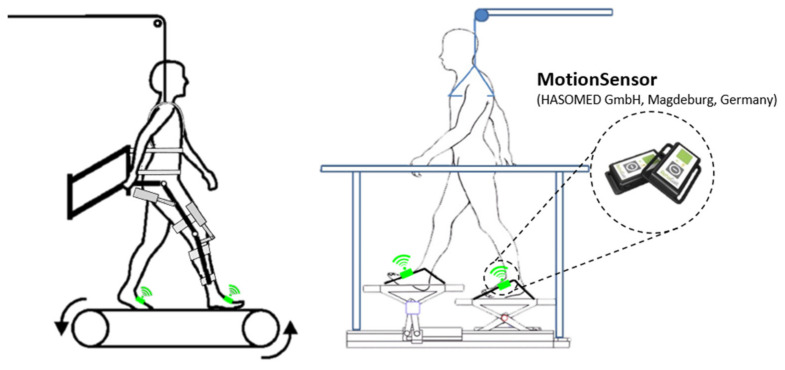
Experimental setup of Lokomat (left) and Lyra (right), adapted from [17].

Prior to the measurement, the therapists adjusted the body weight support system according to the patient’s needs. Two IMUs were attached to the left and right foot of the subject using fixation straps (Figure 2). Afterwards, the exoskeleton of the Lokomat or the foot-plates of the Lyra were adjusted. The amount of body weight support was adjusted according to the subject’s comfort. The recording was started, and the robot-assisted gait therapy was executed according to the rehabilitation routine. The experimental setup is shown in Figure 4.

The data were recorded during the clinical routine. After the recording session, the data was stored as raw data on the host computer using the commercially available software RehaGait Analyzer (HASOMED GmbH, Magdeburg, Germany). The stored raw data was imported into the developed algorithm using the software MATLAB (MathWorks Inc., Massachusetts, MA, USA). The data were stored within the software, and each sample was forwarded individually and sequentially using a custom-made MATLAB function. The function provides a sequential forwarding of the stored samples and thus simulates a faultless real-time Bluetooth connection to the sensors.

### 2.5. Gait Event Detection

**Figure 5:**
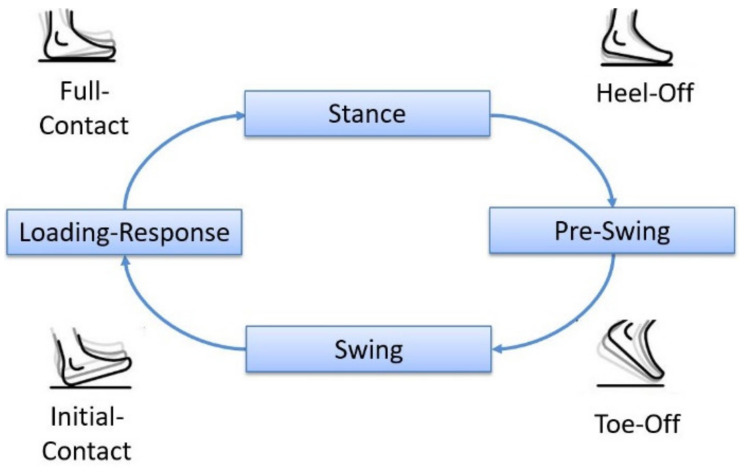
State diagram of gait events, adapted from [19].

The human gait cycle consists of two phases, the stance phase and the swing phase, which are further determined by certain gait events [18]. As not all gait events are reliably detectable with IMUs, the previously developed algorithm was designed to detect four main gait events during robot-assisted gait training [13]. The four main gait events are detected for each IMU according to a state diagram, as can be seen in Figure 5. This state diagram can be assumed since the robots define and limit the movement during the therapy. The algorithm uses linear acceleration data and angular velocity data from the IMUs, which are mounted on the left and right foot of the subject. The extraction of gait events is based on detecting certain characteristics of the data which correspond to a particular event during level-ground walking. Slight variations in the sensor attachment did not influence the experimental setup, due to the implementation of an arbitrary-sensor-alignment algorithm.

**Figure 6:**
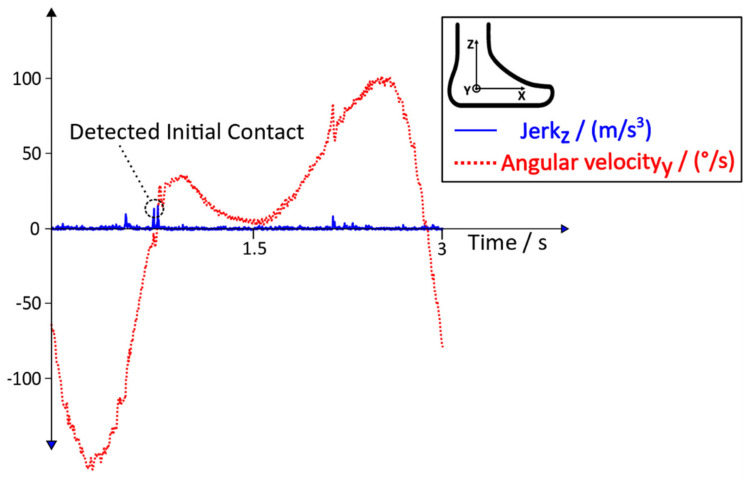
Angular velocity and jerk in the sagittal plane during the robot-assisted gait training of a healthy person, adapted from [13].

During the gait training of healthy subjects, the Initial Contact can be detected due to the distinctive behaviour of the jerk. In Figure 6, the angular velocity (rate of rotational change of the foot) and the jerk (rate of acceleration change of the foot) of a healthy subject during robot-assisted gait training can be seen.

**Figure 7:**
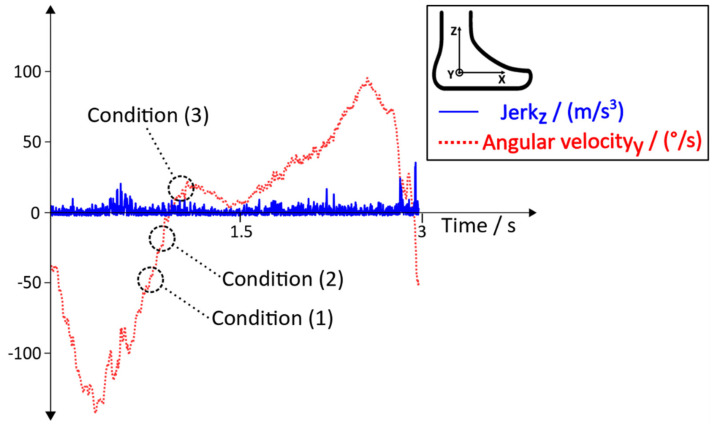
Angular velocity and jerk during the robot-assisted gait training of a stroke patient.

As stated in [13], the linear acceleration data of an IMU during robot-assisted gait training (Figure 4) can differ between the Lokomat and the Lyra. Additionally, patient specific movements or the lack of voluntary movements during training in the Lokomat or the Lyra could influence the behaviour of the acceleration data and the jerk. These additional disruptive factors might lead to an improper detection of the Initial Contact. Thus, additional conditions with the aim to reduce errors and to increase the detection rate were needed. In Figure 6**,** the distinct behaviour of the angular velocity during Initial Contact is shown. Based on this observation, new conditions with the aim to tackle the aforementioned disruptive factors were implemented. The behaviour of the jerk during the robot assisted gait training of a subject who suffered a stroke, and the according angular velocity, including the new conditions, can be seen in Figure 7.

Condition (1) ensures that the angular velocity before detecting an Initial Contact is in a negative range. Condition (1) must be satisfied before the algorithm can check for Condition (2).

(1) $$\omega_{InitialContact\;y_{\left(i\right)}}<0$$

$$\forall \;i\in \left(n\ldots .k\right)$$

Condition (2) checks the angular velocity for a positive slope. As soon as a positive slope is guaranteed, Condition (2) is satisfied and the algorithm is enabled to check for Condition (3).

(2) $$\omega_{InitialContact\;y_{\left(i\right)}}>\omega_{\;InitialContact\;y_{\left(i-1\right)}}$$

$$\forall \;i\in \left(n\ldots .k\right)$$

Condition (3) is the last condition within this sequence, which checks if the angular velocity crosses the zero line and stays at a positive value for a certain number of passed samples.

(3) $$\omega_{InitialContact\;y_{\left(i\right)}}>0$$

$$\forall \;i\in \left(n\ldots .k\right)$$

After the sequence of these conditions is fulfilled, an Initial Contact is detected and the next gait events (Full Contact, Heel Off and Toe Off) can be detected according to the state diagram in Figure 5. The variable n always denotes the current sample of the angular velocity ($\omega_{\mathrm{InitialContact}\;y}$), and k describes a reasonable number of passed samples. These conditions were implemented into the existing algorithm in order to increase the accuracy of the detection of the Initial Contact, with the aim to achieve better robustness and a higher overall detection rate.

#### Error Handling

Erroneous data caused by the unwanted movements of the subject or loose attachment of the sensors can lead to algorithm failure. Additionally, lost data due to connection errors between the sensor and the host computer can lead to the unwanted behavior of the algorithm. In order to minimize the potential risk of algorithm failure, error-handling approaches were implemented. One approach aims to guarantee that the detection of gait events happens according to the defined sequence shown in Figure 5. The gait events must be detected sequentially, starting with the Initial Contact and ending with the Toe-Off. If this motion sequence is not present, the gait event is discarded and the detection process restarts. Another approach aims to minimize algorithm failure by using temporal dependencies between the gait events. After an Initial Contact has been detected, a minimum roll time must elapse before the Heel Off event can be detected. Following the Toe Off event, a minimum swing time must pass before the next Initial Contact can be detected. In addition, a minimum step time must elapse after each Initial Contact before a new step can be detected. The interaction of these error handling methods enables the algorithms to detect errors and discard incorrect events.

### 2.6. Analysing Method

**Figure 8:**
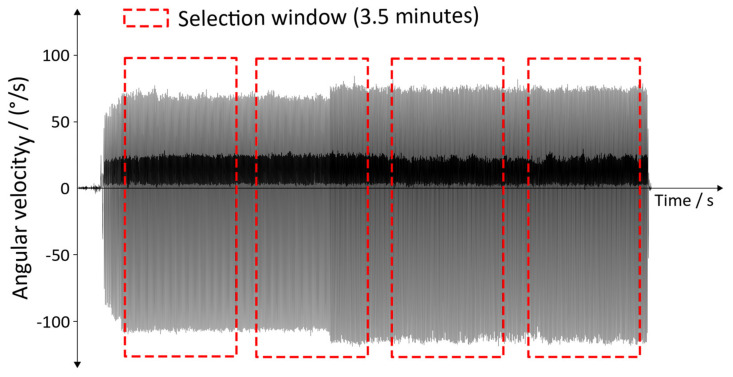
Selection windows for analyzing the data.

The data were analyzed using the aforementioned gait detection algorithm, including the additional conditions mentioned in Section 2.5. Thus, the algorithm extracts four gait events for each IMU. For the analysis, a window of 3.5 min (equaling 105,000 data points) was used. Within this window, the detection rate and the type 1 errors were determined. Depending on the duration of the individual training of the subject, two to four windows were analyzed (Figure 8).

The start of the windows was chosen to be the end of a gait cycle. The according end of the window was determined by the size of the chosen window. This approach was used in order to generate a repeatable and comparable analyzing process within the recording of a subject; additionally, it allows the inter-comparability of the subjects.

The detection rate represents the correctly detected steps, and is defined as follows:

(4) $$Detection\;rate=\frac{100}{\;steps_{reference}}\times steps_{algorithm}$$

Manually counted steps served as a reference ($steps_{reference}$) for defining the detection rate. The steps of the algorithm were calculated by defining the amount of correctly detected steps ($steps_{algorithm}\;$= $steps_{detected}$–$steps_{incorrect\;detected}$). A correctly detected step is only present if all four gait events were detected according to the state diagram in Figure 5. In order to evaluate the robustness and potential flaws of the algorithm, type 1 errors were analyzed. Type 1 errors represent incorrectly detected steps, which can trigger incorrect timing in the electrical stimulation and thus could cause a potential hazard. In contrast with type 1 errors, type 2 errors are not considered hazardous, as steps which are not detected cannot cause incorrect electrical stimulation.

(5) $$Type\;1\;error=\frac{100}{\;steps_{reference}}\times steps_{incorrect\;detected}$$

## 3. Results

**Figure 9:**
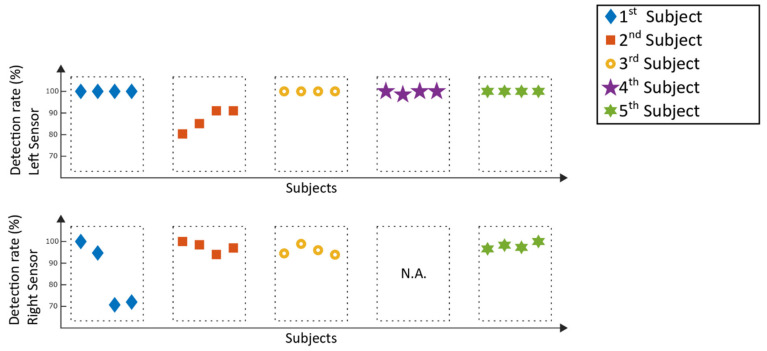
Lyra measurements: detection rates of the selection windows.

**Figure 10:**
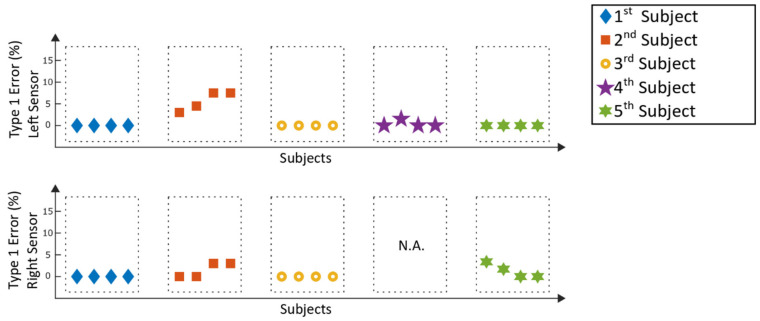
Lyra measurements: type 1 errors of the selection windows.

The following section visualizes the results of the gait event detection algorithm. In Figure 9, the detection rates of the Lyra measurements can be seen, while Figure 10 represents the results of the corresponding type 1 errors. The detection rates represent the percentage of correctly detected steps which could be used for a future trigger of functional electrical stimulation. In contrast to that, type 1 errors represent incorrect detected steps which could be a potentially trigger for hazardous electrical stimulation. Left and right inertial measurement units were analyzed independently from each other.

With the exception of two analyzed selection windows at the right sensor of the 1st subject, with detection rates of 70.7% and 72%, the detection rates for the left and right sensor were above 80%. Type 1 errors from the left sensors were 0% for fifteen out of twenty of the analyzed windows; the remaining five windows suffered type 1 errors below 7.5%. The right sensors suffered type 1 errors below 5%. The mean detection rate of all measurements of the stroke patients was 95.8% ± 7.5%. For type 1 errors, the mean value of all patient data was 1.0% ± 2%. One of the measurements (see Figure 9 and Figure 10) could not be analyzed (N.A.), as the sensor had to be removed during the measurement due to discomfort reported by the patient.

**Figure 11:**
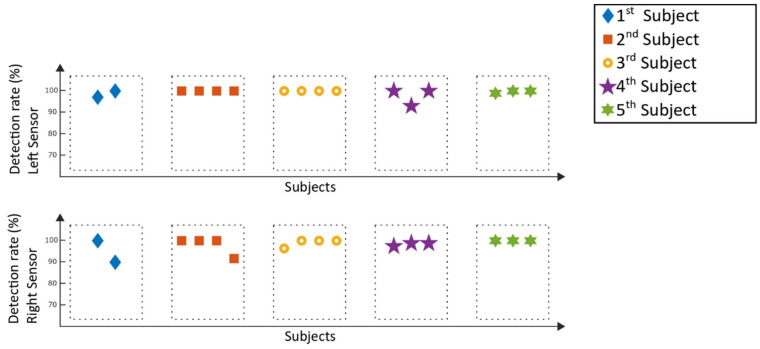
Lokomat measurements: detection rates of the selection windows.

**Figure 12:**
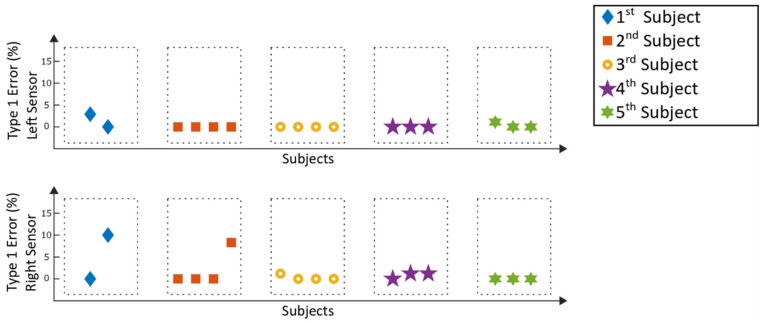
Lokomat measurements: type 1 errors of the selection windows.

The detection rates of the Lokomat measurement are displayed in Figure 11, while Figure 12 represents the corresponding type 1 errors.

For the Lokomat measurements, all detection rates were above 90%, whereas twenty-three out of thirty-two analyzed windows reached a detection value of 100%, which means that every recorded step within those windows could be detected by the algorithm. For twenty-five analyzed windows, type 1 errors of 0% were achieved, five windows stayed below 3% and the remaining two windows had type 1 errors of below 11%. For the Lokomat, the mean detection rate of all patients was 98.7% ± 2.6%, and the according mean type 1 error was 0.9% ± 2.3%.

## 4. Discussion

Within this study, the robustness and feasibility of a gait event detection algorithm for robot-assisted gait training was demonstrated in a clinical setting with patients who suffered a stroke. The patients executed their therapy normally and did not face any disadvantages in the rehabilitation process due to participating in the study. Based on the presented results, the gait event detection algorithm is robust against patient specific movements and the lack of voluntary movements which might influence the behavior of the data during robot-assisted gait training. The newly introduced condition (Section 2.5) is sensitive towards the detection of the Initial Contact, and enables the algorithm to neglect the unwanted detection of the Initial Contact due to the jittering of the jerk (Figure 7). Despite introducing an additional technology (IMUs) to the therapists and the patient, no problems in the application of these devices occurred. The therapist and the patients showed great acceptance and interest in the experimental setup and its potential use within the therapy.

Some subjects were not familiar with robot-assisted gait training, which led to a shorter period of training time. One reason was that the setup time of the gait trainer took longer when the patient was not familiar with the procedure. In addition, longer explanatory conversations were necessary to explain the rehabilitation technique to the patients. Furthermore, exhaustion and the daily constitution of the patients affected the duration of the training. As a result, some analyses were limited to fewer analyzed windows compared to others.

Comparing the results of the detection rates of the Lyra (Figure 9) to the previously conducted study with a healthy adult [13], the detection rates are similar. The same applies to the results achieved with the Lokomat (Figure 11). The type 1 errors for the stroke patients in both gait trainers (Figure 10 and Figure 12) are in a similar range compared to the type 1 errors of a healthy adult as well. Higher type 1 errors of around 11%, as reported in subject one in Figure 12, may have various causes. The sensor might have loosened during the training, causing unwanted sensor behavior. Another reason could be that the settings of the exoskeleton caused an unrecognizable gait cycle, which lead to the improper detection of the gait events and resulting type 1 errors. As high type 1 errors may cause hazardous stimulations, these potential flaws of the experimental setup must be further investigated. Nevertheless, the type 1 errors were low for the majority of the analyzed measurements, providing good results for application in a clinical setting.

As mentioned in Section 2.6, type 2 errors were not reported, because steps that are not detected by the algorithm cannot trigger a stimulation and are therefore not considered hazardous. However, high type 2 errors would influence the percentage of the detection rate and might lead to an inadequate stimulation rate. Thus, type 2 errors should be taken into consideration for the development of the prototype.

The subjects who participated in the study were in their early stage of rehabilitation and new to robot-assisted gait training (Section 2.1). The robots used guide and limit the gait according to their robot specific strategy. Despite the guided movement, the level of gait abnormality might influence the gait pattern, and a hemiplegic leg might be a bigger disruptive factor than a non-hemiplegic leg. In order to evaluate this, a bigger patient population with stricter inclusion criteria should be recruited and analysed for future studies.

The proposed algorithm provides gait-event detection for level-ground walking during robot-assisted gait training. Other gait patterns, such as backwards walking, stairs and slope climbing, must be taken into consideration if the intended use of the algorithm should cover these aspects. So far, gait patterns other than level-ground walking have not been the subject of our research, but might be considered in the future development.

Approaches using techniques like LSTM-DNN (long short-term memory-deep neural network) have reached detection rates of up to 95.1% during level-ground walking on a treadmill [20]. This approach uses the acceleration data from three sensors per leg, located on designated positions (instep, calf and thigh), to detect two gait phase events. Other approaches using deep neural networks on optical motion capture data for the automatic real-time gait event detection reached detection rates of 99% (for foot-contact) and 95% (for foot off) [21]. Approaches using one sensor on the lower back [22], thus minimizing the setup time, do not seem applicable for our purpose, as the bodyweight support system of the gait trainer covers the lower back and limits the movement of the upper body of the subject during the therapy. Comparing the results of our study to the above-mentioned approaches, we achieved similar detection rates using a lightweight, easy approach for practical application during clinical routine. Nevertheless, further studies with a higher number of subjects need to be conducted to prove the results achieved within this study.

As mentioned in Section 2.4, the current system provides a simulated, faultless real-time Bluetooth connection by processing each stored sample individually and sequentially. Real Bluetooth connections may have a delay in transmition. Thus, investigations towards the development of a real Bluetooth connection and their potential problems will be part of the future research. The consideration of the delay of the whole measurement chain will be necessary to evaluate the development of the aforementioned prototype.

The implemented error handling methods to prevent algorithm failure cover a variety of potential errors. Nevertheless, a variation of the experimental setup, such as very high training velocities, could create a combination of events which have not been investigated so far. Despite the adaptability of the algorithm, more data, especially with non-tested velocities, should be recorded in order to prove the effectiveness of the implemented methods and to develop more processing methods to prevent algorithm failure.

In order to be able to generate a repeatable and comparable analysing process, a minimum number of two windows were analysed for each subject. As the shortest usable recording time of a subject was below eight minutes (the first subject in Figure 11 and Figure 12), a time of 3.5 min was chosen for the length of the windows. Robot-assisted gait training provides a repetitive movement. Thus, a longer or shorter time window should not influence the overall detection rate. However, it could happen that a specific movement of a subject, which might influence the data, is within or outside a window and could thus result in changes of the detection rate. Thus, modification of the window length might influence the detection rates, and should be considered in future analysis of the overall detection rate.

Overall, the results are promising, and the application of the algorithm seems feasible and robust when using it in a in a clinical setting for stroke patients. The algorithm is lightweight, and enables an easy setup for usage during a clinical routine. As a next step, the proposed algorithm should be used as a trigger for functional electrical stimulation, with the aim to further enhance the rehabilitation process of stroke patients. Additionally, further processing methods for algorithm failure must be included in order to ensure safe usage with electrical stimulation. In order to establish a prototype of the concept to combine robot-assisted gait training and IMUs to trigger FES, as proposed in [13], further investigations and measurements must be conducted in order to optimize the algorithm and to synchronize the gait events with the stimulation.

## References

[1] Johnson W., Onuma O., Owolabi M., Sachdev S. (2016). Stroke: A global response is needed. Bull. World Health Organ..

[2] Algurén B., Lundgren-Nilsson A., Sunnerhagen K.S. (2010). Functioning of stroke survivors—A validation of the ICF core set for stroke in Sweden. Disabil. Rehabil..

[3] Jørgensen H.S., Nakayama H., Raaschou H.O., Olsen T.S. (1995). Recovery of walking function in stroke patients: The copenhagen stroke study. Arch. Phys. Med. Rehabil..

[4] Dobkin B.H. (2005). Clinical practice. Rehabilitation after stroke. N. Engl. J. Med..

[5] Mehrholz J., Thomas S., Werner C., Kugler J., Pohl M., Elsner B. (2017). Electromechanical-assisted training for walking after stroke. Cochrane Database Syst. Rev..

[6] Hesse S. (2007). Lokomotionstherapie. Ein Praxisorientierter Überblick.

[7] Hidler J.M., Wall A.E. (2005). Alterations in muscle activation patterns during robotic-assisted walking. Clin. Biomech. (Bristol, Avon).

[8] Bruni M.F., Melegari C., Cola M.C., de Bramanti A., Bramanti P., Calabrò R.S. (2018). What does best evidence tell us about robotic gait rehabilitation in stroke patients: A systematic review and meta-analysis. J. Clin. Neurosci..

[9] Laursen C.B., Nielsen J.F., Andersen O.K., Spaich E.G. (2016). Feasibility of Using Lokomat Combined with Functional Electrical Stimulation for the Rehabilitation of Foot Drop. Eur. J. Transl. Myol..

[10] Ng M.F.W., Tong R.K.Y., Li L.S.W. (2008). A pilot study of randomized clinical controlled trial of gait training in subacute stroke patients with partial body-weight support electromechanical gait trainer and functional electrical stimulation: Six-month follow-up. Stroke.

[11] Dohring M.E., Daly J.J. (2008). Automatic synchronization of functional electrical stimulation and robotic assisted treadmill training. IEEE Trans. Neural Syst. Rehabil. Eng..

[12] McCabe J.P. (2008). Feasibility of combining gait robot and multichannel functional electrical stimulation with intramuscular electrodes. JRRD.

[13] Schicketmueller A., Rose G., Hofmann M. (2019). Feasibility of a Sensor-Based Gait Event Detection Algorithm for Triggering Functional Electrical Stimulation during Robot-Assisted Gait Training. Sensors.

[14] Donath L., Faude O., Lichtenstein E., Nüesch C., Mündermann A. (2016). Validity and reliability of a portable gait analysis system for measuring spatiotemporal gait characteristics: Comparison to an instrumented treadmill. J. Neuroeng. Rehabil..

[15] Iosa M., Morone G., Fusco A., Bragoni M., Coiro P., Multari M., Venturiero V., Angelis D., de Pratesi L., Paolucci S. (2012). Seven Capital Devices for the Future of Stroke Rehabilitation. Stroke Res. Treat..

[16] Maranesi E., Riccardi G.R., Di Donna V., Di Rosa M., Fabbietti P., Luzi R., Pranno L., Lattanzio F., Bevilacqua R. (2019). Effectiveness of Intervention Based on End-effector Gait Trainer in Older Patients With Stroke: A Systematic Review. J. Am. Med. Dir. Assoc..

[17] Banz R., Riener R., Lünenburger L., Bolliger M. Assessment of walking performance in robot-assisted gait training: A novel approach based on empirical data. Proceedings of the 2008 30th Annual International Conference of the IEEE Engineering in Medicine and Biology Society.

[18] Neumann D.A., Rowan E.E. (2008). Kinesiology of the musculoskeletal system. Foundations for Physical Rehabilitation.

[19] Seel T., Landgraf L., Escobar V.C., de Schauer T. (2014). Online Gait Phase Detection with Automatic Adaption to Gait Velocity Changes Using Accelerometers and Gyroscopes. Biomed. Tech. (Berl).

[20] Zhen T., Yan L., Yuan P. (2019). Walking Gait Phase Detection Based on Acceleration Signals Using LSTM-DNN Algorithm. Algorithms.

[21] Kidziński Ł., Delp S., Schwartz M. (2019). Automatic real-time gait event detection in children using deep neural networks. PLoS ONE.

[22] McCamley J., Donati M., Grimpampi E., Mazzà C. (2012). An enhanced estimate of initial contact and final contact instants of time using lower trunk inertial sensor data. Gait Posture.

